# Гипергликемия, как побочный эффект алпелисиба: методы профилактики и лечения

**DOI:** 10.14341/probl13337

**Published:** 2023-09-17

**Authors:** Л. М. Кудаева, Е. Е. Кожедуб, В. О. Купрышина, Т. З. Алиев, Е. А. Трошина

**Affiliations:** Московский государственный медико-стоматологический университет имени А.И. Евдокимова; Национальный медицинский исследовательский центр онкологии имени Н.Н. Блохина; Национальный медицинский исследовательский центр онкологии имени Н.Н. Блохина; Национальный медицинский исследовательский центр онкологии имени Н.Н. Блохина; Национальный медицинский исследовательский центр эндокринологии

**Keywords:** алпелисиб, токсичность, эндокринология, гипергликемия, пиоглитазон, метформин, рак

## Abstract

Рак молочной железы (РМЖ) является серьезным заболеванием, считается важной проблемой здравоохранения во всем мире. Распространенность заболевания у женщин, по данным Росстата, составила в РФ — 64 951 случай в 2020 г., (21,7% среди всех видов рака). На долю гормонозависимого эстроген-рецептор-положительного (ЭР+), отрицательного по рецептору эпидермального фактора роста человека 2-го типа (HER2-) метастатического рака молочной железы (мРМЖ) приходится 70% от всех случаев. Около 40% пациентов с ЭР+/HER2- мРМЖ имеют мутации в гене PIK3CA, приводящие к гиперактивации альфа-изоформы (p110α) фосфатидилинозитол-3-киназы (PI3K). Гормональная терапия с использованием или без использования ингибитора циклин-зависимых киназ 4 и 6 (CDK4/6) считается стандартным лечением пациентов с ЭР+/HER2- мРМЖ. Однако приобретенная резистентность к данной терапии остается проблемой. Инновационным методом лечения рака молочной железы является применение таргетных терапевтических агентов, направленных на прямое ингибирование пути PI3K в сочетании с гормонотерапией. Алпелисиб представляет собой PI3Kα-специфический ингибитор.

Гипергликемия выступает в качестве наиболее частого побочного явления при лечении алпелисибом. В настоящее время существует консенсус по профилактике и коррекции гипергликемии у пациентов, получающих терапию препаратом алпелисиб, в котором рекомендуется до начала терапии, с целью диагностики нарушений углеводного обмена и оценки степени риска развития гипергликемии, определять всем пациентам: уровень гликированного гемоглобина (HbA1c), глюкозы плазмы натощак (ГПН), индекс массы тела (ИМТ). А также оценивать такие факторы риска, как наличие семейного анамнеза по сахарному диабету 2 типа (СД2), наличие гестационного СД в анамнезе пациента или факт рождения детей с массой тела более 4 килограммов.

В последнее время активно применяются новые комбинации препаратов для лечения нарушений углеводного обмена, такие как пиоглитазон + метформин. В данной работе обсуждается механизм действия ингибиторов PI3K, новые лечебные комбинации и их нежелательные эффекты, представляется терапевтический опыт.

## Abstract (en)

Breast cancer (BC) is a serious disease and is considered an important health problem worldwide. The prevalence of the disease in women according to Rosstat was 64,951 cases in the Russian Federation in 2020 (21.7% among all types of cancer).  Hormone-dependent estrogen receptor-positive (HR+), human epidermal growth factor receptor type 2 negative (HER2-) metastatic breast cancer (mBC) accounts for 70% of all cases.  About 40% of patients with ER+/HER2- mBC have mutations in the PIK3CA gene, leading to hyperactivation of the alpha isoform (p110α) of phosphatidylinositol 3-kinase (PI3K). Hormonal therapy with or without cyclin-dependent kinase 4 and 6 (CDK4/6) inhibitor is considered the standard treatment for patients with ER+/HER2- mBC. However, acquired resistance to this therapy remains a problem. Innovative methods for the treatment of breast cancer are the use of targeted therapeutic agents aimed at direct inhibition of the PI3K pathway in combination with hormone therapy. Alpelisib is a PI3Kα-specific inhibitor. Hyperglycemia is the most common side effect of alpelisib treatment. Currently, there is a consensus on the prevention and correction of hyperglycemia in patients receiving therapy with alpelisib, which recommends that before starting therapy, in  order to diagnose carbohydrate metabolism disorders and assess the risk of developing hyperglycemia, determine in all patients: the level of glycated hemoglobin (HbA1c), glucose fasting plasma (FPG), body mass index (BMI). And also to evaluate such risk factors as the presence of a family history of type 2 diabetes mellitus (DM 2), the presence of gestational diabetes in the patient’s history, or the fact of the birth of children weighing more than 4 kilograms.

Recently, new combinations of drugs have been actively used to treat disorders of carbohydrate metabolism, such as pioglitazone + metformin. This paper discusses the mechanism of action of PI3K inhibitors, new therapeutic combinations and their undesirable effects, and presents therapeutic experience.

## АКТУАЛЬНОСТЬ

Рак молочной железы (РМЖ) — наиболее распространенное злокачественное новообразование (ЗНО) у женщин в мире. Распространенность заболевания у женщин, по данным Росстата, составила в РФ 64 951 случай в 2020 г., (21,7% среди всех видов рака). На долю гормонозависимого эстроген-рецептор-положительного (ЭР+), отрицательного по рецептору эпидермального фактора роста человека 2 типа (HER2-) метастатического рака молочной железы (мРМЖ) приходится 70% от всех случаев. Около 40% пациентов с ЭР+/HER2- мРМЖ имеют мутации в гене PIK3CA, приводящие к гиперактивации альфа-изоформы (p110α) фосфатидилинозитол-3-киназы (PI3K). Гормональная терапия с использованием или без использования ингибитора циклин-зависимых киназ 4 и 6 (CDK4/6) является стандартным лечением пациентов с ЭР+/HER2- распространенным раком молочной железы [1].

Инновационным методом лечения рака молочной железы является применение таргетных терапевтических агентов, направленных на прямое ингибирование пути PI3K в сочетании с гормонотерапией [2]. Алпелисиб представляет собой PI3Kα-специфический ингибитор, одобренный управлением по контролю качества пищевых продуктов и лекарственных средств (Food and Drug Administration — FDA). Данный ингибитор используется в сочетании с фулвестрантом для лечения женщин в постменопаузе и мужчин с гормоноположительным ЭР+/HER2-, мутированным PIK3CA, мРМЖ [3]. Фулвестрант является антагонистом рецепторов эстрогенов, который в синергии с ингибиторами PI3K блокирует наиболее распространенный путь устойчивости к лечению [4]. Эта комбинация была утверждена на основании результатов рандомизированного двойного слепого плацебо-контролируемого исследования III фазы, проводимого для оценки эффективности и безопасности ингибитора PI3K: алпелисиба в комбинации с фулвестрантом у пациентов с ЭР+/HER2- мРМЖ с мутацией гена PIK3CA (SOLAR-1) [5].

У пациентов с РМЖ с мутированной формой PIK3CA, участвовавших в испытании, выживаемость без прогрессирования была почти удвоена в группе «алпелисиб — фулвестрант» по сравнению с группой «плацебо — фулвестрант» (11,0 вместо 5,7 месяца). Средняя общая выживаемость также была увеличена на 7,9 месяца в алпелисиб-фулвестрантной группе; однако этот анализ не пересекал заранее определенную границу статистической значимости [4].

Одна из важных проблем онкологических и эндокринологических сообществ — побочные проявления на фоне терапии ингибиторами, причем гипергликемия — это наиболее частое побочное проявление при лечении алпелисибом.

Известно, что действие инсулина осуществляется путем связывания с инсулиновым рецептором на поверхности клетки, что в свою очередь активирует внутриклеточные пути, например такие, как путь PI3K (рис. 1). Изоформа p110α PI3K опосредует реакции инсулина в мышцах, печени и жировых тканях [6].

**Figure:**
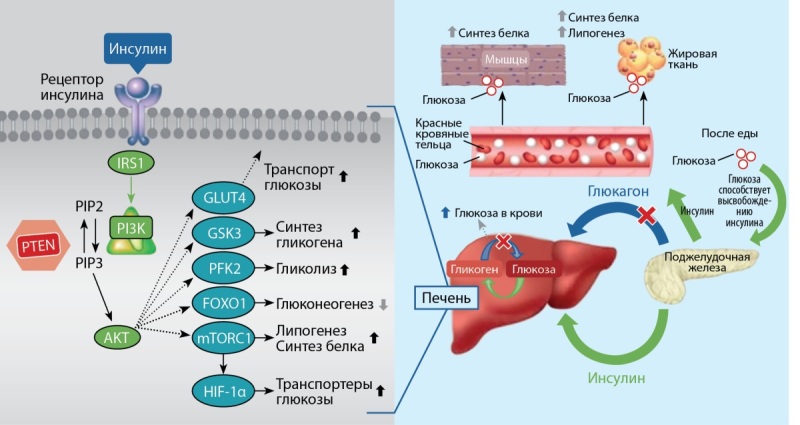
Рисунок 1. Роль инсулина и пути PI3K в нормальном гомеостазе глюкозы [8]. AKT протеинкиназа B, GLUT4 — транспортер глюкозы 4 типа, GS — гликогенсинтаза, IRS1 — субстрат рецептора инсулина 1, PFK2 — фосфофруктокиназа-2, mTORC1 — мишень рапамицинового комплекса 1 у млекопитающих, FOXO1 — фактор транскрипции PI3K фосфоинозитид-3-киназа, PIP2 — фосфатидилинозитол 4, 5-бисфосфат, PIP3 — фосфатидилинозитол 3, 4, 5-трифосфат, PTEN — фосфатаза с двойной субстратной специфичностью, S6K1 — рибосомальная протеинкиназа S6 бета-1, HIF-1a — индуцированный гипоксией фактор 1α. Figure 1. Role of insulin and the PI3K pathway in normal glucose homeostasis. [8] AKT protein kinase B, GLUT4 glucose transporter type 4, GS glycogen synthase, IRS1 substrate of insulin receptor 1, PFK2 phosphofructokinase-2, mTORC1 target of rapamycin complex 1 in mammals, FOXO1 transcription factor PI3K phosphoinositide-3-kinase, PIP2 phosphatidylinositol 4, 5-bisphosphate, PIP3 phosphatidylinositol 3, 4, 5-triphosphate, PTEN phosphatase with double substrate specificity, S6K1 ribosomal protein kinase S6 beta-1, HIF-1a hypoxia-induced factor 1α.

Механизм ингибирования пути PI3K/AKT/mTOR предназначен для вмешательства в рост и выживание раковых клеток; но также ингибирование этого пути приводит к гипергликемии, нарушая внутриклеточный ответ на инсулин, вызывая снижение транспорта глюкозы, снижение синтеза гликогена и увеличение распада глюкозы [7].

Мутация гена PIK3CA является причиной повышенной активации PI3K, которая стимулирует рост и деление раковых клеток [8]. Блокада PI3K с помощью препарата алпелисиб позволяет контролировать этот процесс, однако сопровождается развитием нежелательных явлений (НЯ), наиболее частым из которых отмечается гипергликемия [9]. По данным регистрационного исследования, гипергликемия различной степени выраженности развивается у 64% пациентов, получающих алпелисиб, а у 33% пациентов уровень гликемии достигает 14–28 ммоль/л, что приводит к развитию гиперинсулинемии и может снижать эффективность противоопухолевой терапии [9, 10]. Развитие гипергликемии на фоне применения препарата алпелисиб выступает в качестве физиологической реакции в ответ на блокирование белка PI3K, который регулирует передачу сигнала от рецепторов инсулина [11]. Гипергликемия, развивающаяся на фоне применения препарата алпелисиб, носит характер временного нежелательного явления, которое разрешается после отмены лечения, не формируя хронического нарушения метаболизма.

Согласно консенсусу по профилактике и коррекции гипергликемии, у пациентов, получающих терапию препаратом алпелисиб, пациент может быть отнесен к группе низкого риска при соответствии всех перечисленных критериев: ИМТ<25 кг/м², ГПН<6,1 ммоль/л, HbA1c<5,7%. Пациент может быть отнесен к группе умеренного риска при сочетании нормального уровня ГПН (<6,1 ммоль/л) с HbA1c 5,7–5,9% и/или наличием избыточной массы тела и хотя бы одного из дополнительных факторов риска. К группе высокого риска относят пациентов с избыточной массой тела или ожирением в сочетании с уровнем HbA1c 6,0–6,4% и/или диагностированным ранее преддиабетом (нарушенная гликемия натощак, нарушенная толерантность к глюкозе) [3].

В настоящее время актуально применение инсулин-сенситайзеров — к которым относят группы препаратов бигуаниды (метформин) и тиазолидиндионы (пиоглитазон), — с целью борьбы с инсулинорезистентностью, вызванной терапией алпелисибом. Взаимодополняющие действия пиоглитазона и метформина дают возможность лучше контролировать уровень глюкозы в крови. Эффективность заключается не только в улучшении контроля глюкозы, но и в снижении метаболического риска.

Мы представляем клинический случай развития гипергликемии у пациентки 60 лет с РМЖ на фоне специального лечения.

## ОПИСАНИЕ СЛУЧАЯ

Пациентка Н. 60 лет. Впервые в 2011 г. поступила с предварительным диагнозом: «рак молочной железы» для дообследования и проведения специального лечения.

Анамнез жизни не отягощен. Семейный анамнез — отягощенный: сахарный диабет 2 типа у отца.

Пациентке, по результатам комплексного обследования, выставлен заключительный клинический диагноз: «рак левой молочной железы T1N2M0» и инициировано комплексное лечение в объеме: оперативное лечение — субтотальная радикальная резекция левой молочной железы (гистологическое заключение: инфильтрирующий дольковый рак); 5 курсов полихимиотерапии (ПХТ) по схеме CAF (циклофосфамид, метотрексат, 5-фторурацил); дистанционная лучевая терапия на шейно-надключичные л/у, парастернальные подмышечные л/у; суммарная очаговая доза (СОД) — 45 Гр, на ложе удаленной опухоли СОД — 50 Гр. По окончании ранее описанного комплексного лечения активирована антигормональная терапия препаратом Летрозол до 2016 г. включительно.

В начале 2021 г., по результатам контрольного обследования, у пациентки диагностировано прогрессирование основного заболевания в виде метастатического поражения костной структуры 12 грудного позвонка (согласно результатам ПЭТ КТ). Выполнено оперативное вмешательство в объеме: трепан-биопсия 12 грудного позвонка. По данным гистологического (Г/И) и иммуногистохимического (ИГХ) заключений: метастаз рака молочной железы, РЭ 4 балла, РП 0 баллов, HER2/neu отриц, Ki-67 4%.

Согласно результатам Г/И и ИГХ, с середины 2021 г., инициирована вторая линия специфической терапии в объеме: палбоциклиб, летрозол. При динамическом контроле (ПЭТ КТ от сентября 2022 г.) — прогрессирование заболевания в виде появления очагов в печени, новых очагов — в телах позвонков, подвздошных костях, крестце.

С октября 2022 г. начата терапия антиэстрогенным препаратом с противоопухолевым действием — фулвестрант, с декабря терапия дополнена PI3Kα-специфическим ингибитором — алпелисиб в дозировке 300 мг/сут. На момент лечения у пациентки отмечалось нарушение гликемии натощак в течение последних 2 лет (гликемия в диапазоне 6-6,5 ммоль/л). Постоянная гипогликемическая терапия не проводилась. Из объективного статуса известно: масса тела — 66 кг, рост — 158 см, ИМТ — 26,4 кг/м². Из лабораторных данных отмечается гликированный гемоглобин — 6,2%. Пациентке начата терапия метформином пролонгированного действия в дозировке 2000 мг/сут, алоглиптином — 25 мг/сут, пиоглитазоном — 30 мг/сут. Также прописана диета с ограничением углеводов с высоким гликемическим индексом и умеренная физическая активность. Повышение гликемии отмечалось со второго дня приема алпелисиба, препрандиальная гликемия до 7,5 ммоль/л, постпрандиальная гликемия до 11 ммоль/л (рис. 2).

**Figure:**
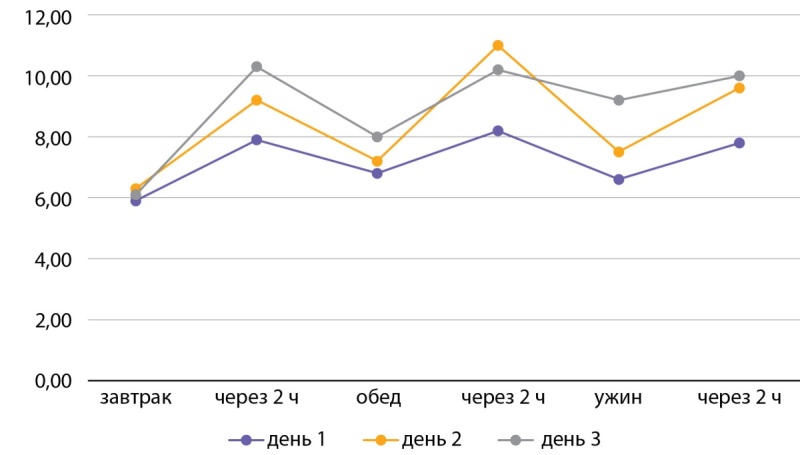
Рисунок 2. Контроль глюкозы в крови на фоне терапии (первые 72 часа). Figure 2. Blood control for immune therapy (first 72 hours).

На 7-е сутки терапии отмечалась стойкая гипергликемия, максимально до 17,5 ммоль/л (рис. 3).

**Figure:**
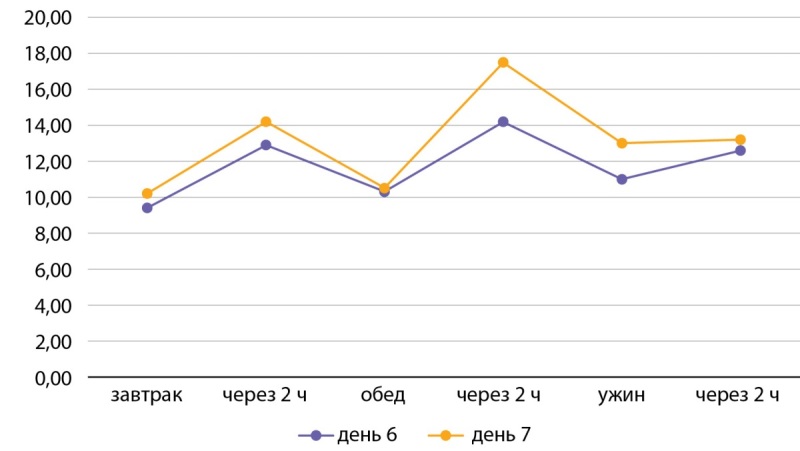
Рисунок 3. Контроль глюкозы в крови на фоне терапии (на 6-е и 7-е сутки). Figure 3. Blood control for immune therapy (for 6 and 7 days).

В гипогликемическую терапию добавлен дапаглифлозин 10 мг/сут. На фоне данной терапии отмечалась положительная динамика к 10–11-м суткам в виде снижения гликемии (рис. 4).

**Figure:**
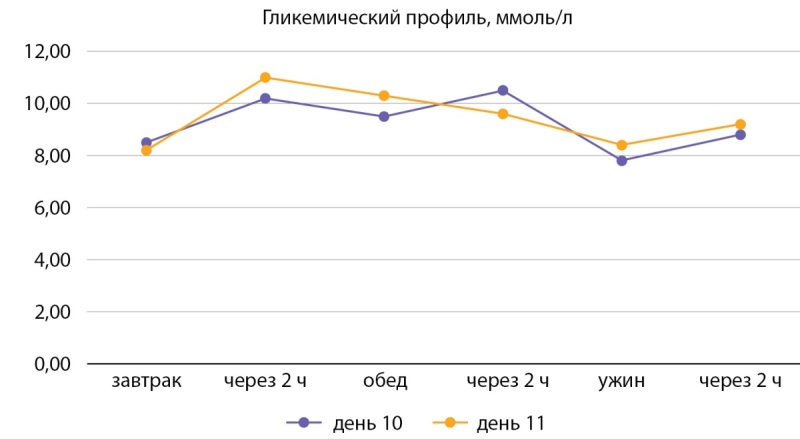
Рисунок 4. Контроль глюкозы в крови на фоне терапии (на 10-е и 11-е сутки). Figure 4. Blood control for immune therapy (for 10 and 11 days).

На 15-е сутки лечения алпелесибом пациентка стала отмечать резко сниженный аппетит и кожный зуд. Для контроля за кетонурией ежедневно использовались тест-полоски. На 17-е сутки регистрировалось ухудшение состояния в виде усиления кожного зуда и появления болей в области живота. По результатам обследований у пациентки диагностирован холестатический синдром (ГГТ — 125 ед/л, ЩФ — 150 ед/л, АЛТ — 50 ед/л, АСТ — 15 ед/л), в клиническом анализе мочи определялись кетоновые тела (+++). Терапия алпелисибом временно прекращена. Дапаглифлозин, алоглиптин и пиоглитазон отменены, в терапию добавлен инсулин изофан (Протафан НМ) по 10 ед. 2 раза в сутки, инсулин короткого действия аспарт (новорапид) по 5–7 ед. перед приемами пищи и дополнительно — на коррекцию гипергликемии. Пациентка осмотрена гастроэнтерологом, назначена терапия урсодезоксихолевой кислотой.

Через 10 дней от начала инсулинотерапии отмечается стабилизация показателей гликемии, при контрольном анализе мочи кетоны не обнаружены. Возобновлена терапия алпелисибом в редуцированной дозе 250 мг/сут. Гипогликемическая терапия: инсулин изофан 12 ед. 2 раза в день, дапаглифлозин 10 мг/сут, метформин пролонгированного действия 2000 мг/сут, алоглиптин 25 мг/сут, пиоглитазон 30 мг/сут. Целевые показатели гликемии достигнуты при общей суточной дозе инсулина изофан 24 ед/сут. Препрандиальная гликемия до 8 ммоль/л, постпрандиальная гликемия до 11 ммоль/л (рис. 5).

**Figure:**
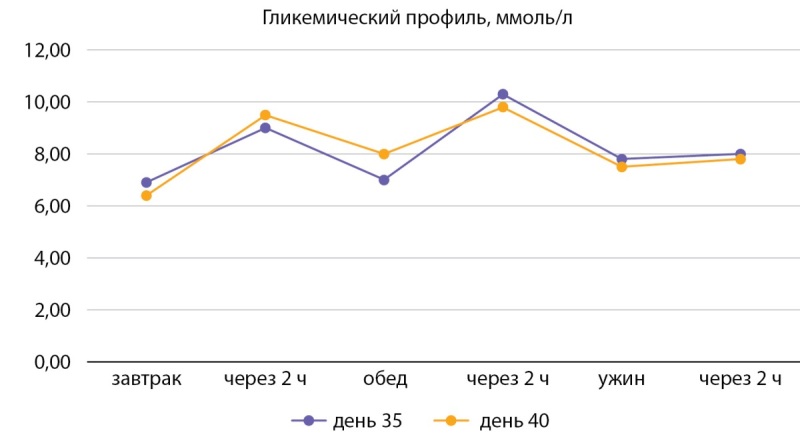
Рисунок 5. Контроль глюкозы в крови на фоне терапии (на 35–40-е сутки). Figure 5. Blood control for immune therapy (for 35–40 days).

На фоне совместного использования пиоглитазона и инсулина средней длительности отечный синдром не отмечался. Кетоновые тела в динамике отрицательные. По данным контрольных инструментальных исследований (ПЭТ КТ) от марта 2023 г.: положительная динамика в виде уменьшения метаболической активности очагов в печени. Гликированный гемоглобин через 3 месяца лечения составлял 8,5%, что свидетельствует о декомпенсации углеводного обмена. В настоящий момент пациентка продолжает специальное лечение алпелесибом 250 мг/сут (редуцированная доза); гипогликемическая терапия в объеме: инсулин изофан 24 ед/сут, дапаглифлозин 10 мг/сут, метформин пролонгированного действия 2000 мг/сут, алоглиптин 25 мг/сут, пиоглитазон 30 мг/сут.

## ОБСУЖДЕНИЕ

В исследовании SOLAR-1 более высокая частота гипергликемии наблюдалась у пожилых пациентов (≥75 лет) и пациентов с избыточным весом и ожирением на базовом уровне, получавших алпелисиб [12].

В настоящее время существует консенсус по профилактике и коррекции гипергликемии у пациентов, получающих терапию препаратом алпелисиб, составленный экспертами Российской ассоциации эндокринологов и Российского общества клинической онкологии. В нем рекомендуется до начала терапии, с целью диагностики нарушений углеводного обмена и оценки степени риска развития гипергликемии, определять всем пациентам: уровень гликированного гемоглобина (HbA1c), глюкозы плазмы натощак (ГПН), индекс массы тела (ИМТ). А также оценивать такие факторы риска, как наличие семейного анамнеза по СД2, наличие гестационного СД в анамнезе пациента или факт рождения детей с массой тела более 4 килограммов.

Согласно данному консенсусу выделяют 3 группы пациентов.

Группа низкого риска: ИМТ<25 кг/м², ГПН<6,1 ммоль/л, HbA1c<5,7%. Группа умеренного риска: ГПН (<6,1 ммоль/л), HbA1c 5,7–5,9% и/или наличие избыточной массы тела или хотя бы одного из дополнительных факторов риска. К группе высокого риска относят пациентов с избыточной массой тела или ожирением в сочетании с уровнем HbA1c 6,0–6,4% и/или диагностированным ранее предиабетом (нарушенная гликемия натощак, нарушенная толерантность к глюкозе). В качестве профилактики рекомендуется диета с исключением легкоусвояемых углеводов и умеренным ограничением медленноусвояемых углеводов, а также прием метформина в дозе, соответствующей группе риска.

Начальная доза метформина в группе низкого риска при ГПН выше 6,5 ммоль/л — 500 мг/сут с постепенным повышением дозы до 2000 мг/сут. Для группы умеренного риска рекомендуется профилактический прием метформина в суточной дозе 500–1000 мг до начала терапии алпелисибом, при ГПН>6,5 ммоль/л на фоне лечения —увеличение дозы до 2000 мг/сут, при необходимости назначается комбинация с другими сахароснижающими препаратами. В группе высокого риска всем пациентам до начала терапии алпелисибом назначается метформин в дозе 2000 мг/сут. При ГПН>7 ммоль/л рекомендуется комбинация метформина с другими сахароснижающими препаратами.

Также согласно консенсусу, в качестве препаратов 2-й линии сахароснижающей терапии рекомендуется назначать ингибиторы натрий-глюкозного котранспортера 2-го типа — НГЛТ-2. При необходимости усиления сахароснижающей терапии рекомендуется назначение комбинации 3–5 препаратов, предпочтительным является выбор лекарственных средств, улучшающих чувствительность к инсулину [2].

Blow T., Hyde P.N., Falcone J.N. et al. в 2021 г. определили 2 стратегии, которые эффективно отменили эти целевые неблагоприятные эффекты у пациентов: низкоуглеводная диета, благодаря которой истощаются запасы гликогена в печени и повышение выведения глюкозы мочой с помощью ингибиторов НГЛТ-2. В данной работе коллеги описали 3 клинических случая — пациенты с ЭР+/HER2−, мРМЖ, которые использовали в лечении алпелисиб, вызвавший гипергликемию. В первом клиническом случае пациент с сахарным диабетом первого типа держал под контролем сахар с помощью системы непрерывного мониторинга глюкозы (НМГ) и подкожной инсулиновой помпы. Благодаря полученным данным непрерывного мониторирования, удалось зафиксировать влияние алпелисиба на гомеостаз глюкозы. Наблюдалась острая, обратимая и глубокая резистентность к инсулину при введении алпелисиба, а также суточная потребность в инсулине увеличилась в 4,5 раза. Но если придерживаться низкоуглеводной диеты, можно понизить потребность. Во втором клиническом случае у пациента в анамнезе — нарушение гликемии натощак, и с началом приема алпелисиба развилась неконтролируемая гипергликимия. Для коррекции глюкозы был использован метформин и сенсибилизатор инсулина — пиоглитазон. Между тем скорректировать уровень глюкозы удалось только после назначения ингибитора НГЛТ-2, что позволило пациенту продолжить лечение алпелисибом еще 88 дней. У пациента с сахарным диабетом второго типа, описанного в третьем киническом случае, нарастала гипергликемия, несмотря на то что он придерживался низкоуглеводной диеты. Для регуляции глюкозы был использован ингибитор НГЛТ-2, и значения быстро скорректировались [13].

Инсулинорезистентность влечет за собой многочисленные нарушения в системах организма, участвующих в гомеостазе глюкозы. Таким образом, терапия, понижающая уровень глюкозы и устраняющая резистентность к инсулину, должна включать комбинации механизмов действия. Различие заключается в разном воздействии на гомеостаз печени и периферической глюкозы. Механизм действия метформина направлен на повышение скорости утилизации глюкозы мышцами за счет активации анаэробного гликолиза, а также угнетение глюконеогенеза, а пиоглизатон улучшает чувствительность к инсулину непосредственно за счет активации рецепторов PPARγ (peroxisome proliferator-activated receptor (рецепторы, активируемые пероксисомными пролифераторами)), которые экспрессируются в адипоцитах, печени и скелетных мышцах. Их активация обуславливает повышение экспрессии транспортеров глюкозы GLUT-1 и GLUT-4 на мембранах клеток печени и скелетной мускулатуры, что приводит к увеличению поглощения свободных жирных кислот и отложению их в подкожной жировой ткани [14].

Пиоглитазон в комбинации с инсулином может вызывать отечный синдром, это связанно с тем, что препарат стимулирует инсулиновые рецепторы PPARγ, которые при взаимодействии с инсулином вызывают задержку свободной жидкости в организме. В нашем клиническом случае у пациентки не наблюдался отечный синдром, ввиду выраженной инсулинорезистентности, а также интенсивной терапии ингибиторами НГЛТ-2, которые обладают диуретическим свойством.

Таким образом, алгоритм лечения гипергликемии, вызванной алпелисибом, следующий: препаратами первой линии являются сенситайзеры рецепторов инсулина — метформин и пиоглитазон, так как основная причина гипергликемии при лечении алпелисибом — резистентность к инсулину, ввиду того, что блокируется сам путь PI3K, который регулирует передачу сигнала от рецепторов инсулина. В качестве второй линии — ингибиторы НГЛТ-2, поскольку эти препараты действуют инсулин-независимо и посредством угнетения реабсорбции глюкозы в проксимальных почечных канальцах увеличивают ее экскрецию с мочой, в результате за счет гликозурии снижается уровень глюкозы в плазме. Однако следует контролировать кетоновые тела в моче по причине возможного развития кетоацидоза. Препараты, стимулирующие выработку инсулина, либо непосредственно сам инсулин не рекомендуется использовать, так как снижается эффективность алпелисиба. Но в некоторых случаях, например как в нашем, применение инсулина необходимо, чтобы вывести пациента из состояния кетоацидоза.

Начальная доза алпелисиба составляет 300 мг/сут. При необходимости она может быть снижена до 250, а затем до 200 мг/сут. Временная приостановка приема алпелисиба и возобновление приема в меньшей дозе из-за гипергликемии требуется при: развитии гипергликемии 3-й и 4-й степени (уровень ГПН>13,9 ммоль/л и ГПН>27,8 ммоль/л), а также при невозможности компенсировать показатели углеводного обмена на фоне комбинированной сахароснижающей терапии, включая инсулинотерапию. Алпелисиб повторно инициируется в более низкой дозе при достижении уровня ГПН<8,9 ммоль/л. Отмена алпелисиба из-за гипергликемии требуется при развитии диабетического кетоацидоза или невозможности компенсировать показатели углеводного обмена на фоне комбинированной сахароснижающей терапии, включая инсулинотерапию, после снижения дозы алпелисиба до 200 мг/сут [3].

В связи с основным побочным проявлением, гипергликимией, требуется тщательное изучение терапевтических комбинаций с алпелисибом, что позволит достичь значительной эффективности от антионкогенных свойств препарата без необходимости прерывания терапии.

## ЗАКЛЮЧЕНИЕ

Терапия осложнений на фоне лечения алпелисибом РМЖ является одной из важных проблем, требующей дальнейшего изучения и проведения дополнительных перспективных клинических исследований для определения оптимальной стратегии лечения гипергликемии в целях предотвращения снижения дозы алпелисиба и прекращения лечения. Новые комбинации ингибиторов, используемые в терапии пациентов с раком молочной железы, могут открыть широкие терапевтические перспективы, особенно в случаях резистентности к гормональной монотерапии.

## ДОПОЛНИТЕЛЬНАЯ ИНФОРМАЦИЯ

Источники финансирования. Работа выполнена по инициативе авторов без привлечения финансирования.

Конфликт интересов. Авторы декларируют отсутствие явных и потенциальных конфликтов интересов, связанных с содержанием настоящей статьи.

Участие авторов. Кудаева Л.М. — обзор публикаций по теме статьи, анализ полученных данных, написание текста статьи; Кудаева Л.М., Кожедуб Е.Е. — разработка дизайна статьи, подготовка списка литературы, составление резюме; Купрышина В.О., Кожедуб Е.Е., Алиев Т.З. — обзор публикаций по теме статьи, написание текста статьи, изучение разных групп пациентов, анализ полученных данных, подготовка списка литературы; Трошина Е.А.— научное редактирование статьи.

Все авторы одобрили финальную версию статьи перед публикацией, выразили согласие нести ответственность за все аспекты работы, подразумевающую надлежащее изучение и решение вопросов, связанных с точностью или добросовестностью любой части работы.

Согласие пациента. Информированное согласие от пациента добровольно получено и подписано на публикацию персональной медицинской информации в обезличенной форме (именно в этом журнале).

Благодарности. Выражаем благодарность своему научному руководителю Трошиной Екатерине Анатольевне за ценные советы при планировании научной работы и рекомендации по оформлению статьи.

## References

[cit1] Kaprin A.D., Starinskii V.V., Petrova G.V. Zlokachestvennye novoobrazovaniya v Rossii v 2020 godu (zabolevaemost' i smertnost'). M.: MNIOI im. P.A. Gertsena filial FGBU «NMIRTs» Minzdrava Rossii, 2020

[cit2] Stenina M.B., Zhukova L.G., Koroleva I.A. i dr. Prakticheskie rekomendatsii po lekarstvennomu lecheniyu raka molochnoi zhelezy. Zlokachestvennye opukholi. 2020. — 10(3s2). https://rosoncoweb.ru/standarts/RUSSCO/2020/2020-09.pdf

[cit3] Mazurina Natalya V., Artamonova Elena V., Beloyartseva Maria F., Volkova Ekaterina I., Ganshina Inna P., Troshina Ekaterina A., Tjulandin Sergey A., Chubenko Viacheslav A. (2021). The consensus on the prevention and correction of hyperglycemia in patients with HR+ HER2- metastatic breast cancer treated with alpelisib. Journal of Modern Oncology.

[cit4] André F., Ciruelos E.M., Juric D., Loibl S., Campone M., Mayer I.A., Rubovszky G., Yamashita T., Kaufman B., Lu Y.-S., Inoue K., Pápai Z., Takahashi M., Ghaznawi F., Mills D., Kaper M., Miller M., Conte P.F., Iwata H., Rugo H.S. (2020). Alpelisib plus fulvestrant for PIK3CA-mutated, hormone receptor-positive, human epidermal growth factor receptor-2–negative advanced breast cancer: final overall survival results from SOLAR-1. Annals of Oncology.

[cit5] Miller Todd W., Balko Justin M., Arteaga Carlos L. (2011). Phosphatidylinositol 3-Kinase and Antiestrogen Resistance in Breast Cancer. Journal of Clinical Oncology.

[cit6] Goncalves Marcus D., Farooki Azeez (2022). Management of Phosphatidylinositol-3-Kinase Inhibitor-Associated Hyperglycemia. Integrative Cancer Therapies.

[cit7] Goldman Jonathan W., Mendenhall Melody A., Rettinger Sarah R. (2016). Hyperglycemia Associated With Targeted Oncologic Treatment: Mechanisms and Management. The Oncologist.

[cit8] Semiglazova T.Yu. Semiglazova, Semiglazov V.V. Semiglazov, Klimenko V.V. Klimenko, Brish N.A. Brish, Alekseeva Yu.V. Alekseeva, Klyuge V.A. Klyuge, Krutov A.A. Krutov, Paltuev R.M. Paltuev, Kasparov B.S. Kasparov, Krivorotko P.V. Krivorotko, Semiglazov V.F. Semiglazov (2020). Alpelisib for the treatment of HR+HER2-metastatic breast cancer in patients with the PIK3CA mutation: results of the SOLAR-1 study. Pharmateca.

[cit9] André Fabrice, Ciruelos Eva, Rubovszky Gabor, Campone Mario, Loibl Sibylle, Rugo Hope S., Iwata Hiroji, Conte Pierfranco, Mayer Ingrid A., Kaufman Bella, Yamashita Toshinari, Lu Yen-Shen, Inoue Kenichi, Takahashi Masato, Pápai Zsuzsanna, Longin Anne-Sophie, Mills David, Wilke Celine, Hirawat Samit, Juric Dejan (2019). Alpelisib for PIK3CA-Mutated, Hormone Receptor–Positive Advanced Breast Cancer. New England Journal of Medicine.

[cit10] Rugo H.S., André F., Yamashita T., Cerda H., Toledano I., Stemmer S.M., Jurado J.C., Juric D., Mayer I., Ciruelos E.M., Iwata H., Conte P., Campone M., Wilke C., Mills D., Lteif A., Miller M., Gaudenzi F., Loibl S. (2020). Time course and management of key adverse events during the randomized phase III SOLAR-1 study of PI3K inhibitor alpelisib plus fulvestrant in patients with HR-positive advanced breast cancer. Annals of Oncology.

[cit11] Świderska Ewa, Strycharz Justyna, Wróblewski Adam, Szemraj Janusz, Drzewoski Józef, Śliwińska Agnieszka (2019). Role of PI3K/AKT Pathway in Insulin-Mediated Glucose Uptake. Blood Glucose Levels.

[cit12] Rugo H.S., André F., Yamashita T., Cerda H., Toledano I., Stemmer S.M., Jurado J.C., Juric D., Mayer I., Ciruelos E.M., Iwata H., Conte P., Campone M., Wilke C., Mills D., Lteif A., Miller M., Gaudenzi F., Loibl S. (2020). Time course and management of key adverse events during the randomized phase III SOLAR-1 study of PI3K inhibitor alpelisib plus fulvestrant in patients with HR-positive advanced breast cancer. Annals of Oncology.

[cit13] Blow Tahj, Hyde Parker N., Falcone John N., Neinstein Aaron, Vasan Neil, Chitkara Ritika, Hurd Maurice A., Sardesai Sagar, Lustberg Maryam B., Flory James H., Volek Jeff S., Goncalves Marcus D. (2021). Treating Alpelisib-Induced Hyperglycemia with Very Low Carbohydrate Diets and Sodium-Glucose Co-Transporter 2 Inhibitors: A Case Series. Integrative Cancer Therapies.

[cit14] Pesheva Ekaterina D., Fadeev Valentin V. (2021). Pioglitazone is a forgotten hypoglycemic drug with proven cardioprotective and nephroprotective properties. Consilium Medicum.

